# P-2106. Streamlining Care Delivery in the Pediatric Emergency Department Using Rapid Point-Of-Care Molecular Diagnostics For Group A Streptococcus: A Randomized Controlled Trial

**DOI:** 10.1093/ofid/ofae631.2262

**Published:** 2025-01-29

**Authors:** Jeffrey Pernica, April J Kam, Fiona Smaill, Joycelyne Ewusie, Sarah Khan, Shakeap Elliott, Melani Sung, Quynh Doan, Marek Smieja, Lehana Thabane, David Goldfarb

**Affiliations:** Department of Pediatrics, McMaster University, Hamilton, Ontario, Canada; McMaster University, Hamilton, Ontario, Canada; McMaster University, Hamilton, Ontario, Canada; St Joseph's Healthcare Hamilton, Hamilton, Ontario, Canada; McMaster University, Hamilton, Ontario, Canada, Hamilton, Ontario, Canada; Hamilton Health Sciences, Hamilton, Ontario, Canada; Hamilton Health Sciences, Hamilton, Ontario, Canada; University of British Columbia, Vancouver, British Columbia, Canada; McMaster University, Hamilton, Ontario, Canada; McMaster University, Hamilton, Ontario, Canada; University of British Columbia, Vancouver, British Columbia, Canada

## Abstract

**Background:**

As per IDSA guidelines, throat swab testing for group A Streptococcus is important to ensure appropriate antimicrobial use for children with pharyngitis that is not obviously viral. Bacterial culture is the diagnostic reference standard but takes days to result. With culture, physicians often initially provide a prescription that is only to be filled later on if GAS testing is positive. Unfortunately, this strategy can result in inappropriate treatment if parents of children with negative tests fill the script anyway, or if parents of children with positive tests cannot be contacted later. Point-of-care (POC) antigen testing in children is specific but not sensitive, so a negative test still requires culture verification, which limits its overall usefulness. POC molecular GAS testing is as sensitive as culture, and provides a result in minutes, which simplifies prescribing.

ED physician satisfaction at enrolment

**Figure:**
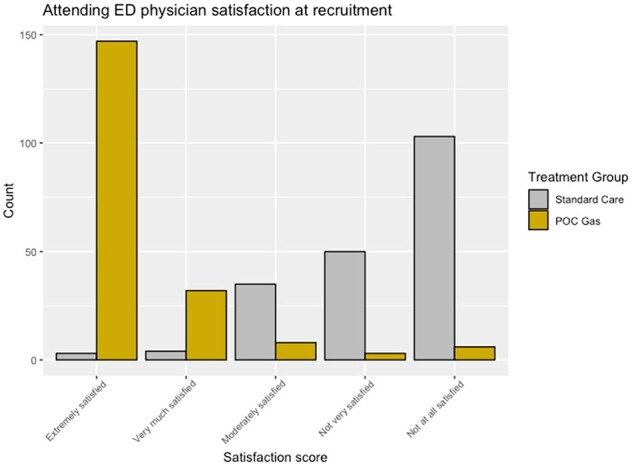

Satisfaction was captured using a 5-point Likert scale on the day of enrolment.

**Methods:**

A randomized controlled trial was done in a Canadian children’s emergency department (ED). Children aged >3 y with throat swabs ordered to diagnose GAS pharyngitis were eligible. Participants were randomized 1:1 to either the IDNOW Strep A2 (intervention) or bacterial culture (control). The primary outcome was appropriate treatment, ie taking antibiotics if GAS-positive or *not* taking antibiotics if GAS-negative. Secondary outcomes included physician and parent satisfaction and time to symptom resolution.

Caregiver satisfaction at enrolment

**Figure:**
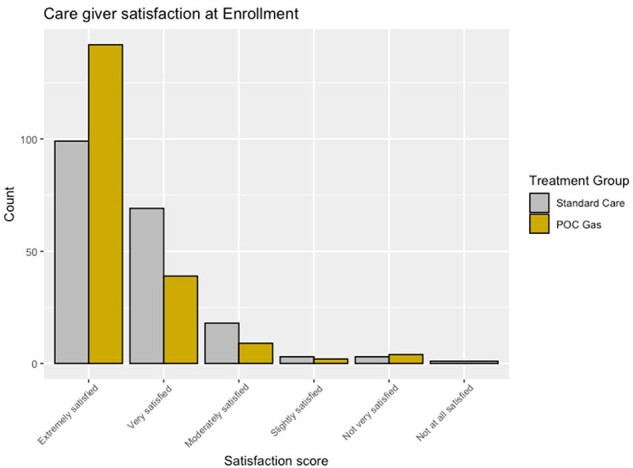

Satisfaction was captured using a 5-point Likert scale on the day of enrolment.

**Results:**

392 participants were recruited from Jan 2021 – Mar 2024. Intervention and control groups were similar in terms of mean age (8.2 vs 8.3 y) and mean MacIsaac score (both 3.3); 29% of the intervention group and 41% of the control group were GAS-positive. Participants in the intervention group were significantly more likely to receive appropriate antibiotics (90.8% vs 76.3%, OR 3.1 [95%CI 1.7-5.7], p< 0.001). ED physicians (pOR 147, p< 0.001) and parents (pOR 2.4, p< 0.001) were also much more likely to be satisfied with POC molecular testing. There were no statistically significant differences in time to resolution of symptoms, though comparisons favoured the intervention.

**Conclusion:**

In the largest RCT of molecular GAS testing yet done, the intervention was associated with substantial improvements in appropriate antibiotic prescribing and was clearly preferred by both ED physicians and parents.

**Disclosures:**

Jeffrey Pernica, MD, MSc, FRCPC, DTMH, MedImmune: Grant/Research Support|Merck: Grant/Research Support

